# Curricular and pedagogical approaches for physical activity prescription training: a mixed-methods study of the “Exercise is Medicine” workshops in Colombia

**DOI:** 10.1186/s12909-023-04999-3

**Published:** 2024-01-22

**Authors:** Diana C. Páez, Johanna Flórez, María Teresa Gómez, Daniel García, Carlos M. Arango-Paternina, John Duperly

**Affiliations:** 1 School of Medicine, Universidad de los Andes, Carrera 1, #18a‐12, Bogotá, Colombia; 2 Centro de Investigación y Formación en Educación, Universidad de los Andes, Carrera 1, #18a‐12, Bogotá, Colombia; 3 Instituto Universitario de Educación Física y Deporte, Universidad de Antioquia, Calle 74 # 70 - 59, Medellín, Colombia; 4 Institute of Exercise Medicine and Rehabilitation, Fundación Santa Fe de Bogotá, Cra. 7 #117 -15, Bogotá, Colombia

**Keywords:** Educational workshop, Continuing medical education, Curricula, Physical activity, Exercise is medicine, Physicians

## Abstract

**Background:**

The physical activity (PA) prescription workshop for physicians, through the global health initiative “Exercise is Medicine” (EIM), has trained more than 4000 health care professionals (HCPs) in Latin America. It has shown to be effective in increasing PA prescription knowledge and awareness among HCPs. The purpose of this paper is to evaluate the curricular and pedagogical approach used by EIM Colombia at the PA prescription workshops implemented between 2014 and 2015.

**Methods:**

A mixed methods study, with a sequential explanatory design was implemented among a convenience sample of HCPs attending twenty-six PA prescription workshops. HCPs health status, PA personal habits, and medical practices were collected using a questionnaire at baseline among 795 participants (pre-test measurement), and subsequently quantitatively analyzed. A workshop satisfaction survey was administered after the completion of the workshop among 602 HCPs. The curricular and pedagogical approach of the workshop, the designers’ and students’ contextual factors, and perceptions about the workshop were measured using qualitative methods (analysis of the procedures manual, two workshop observations, three semi-structured interviews, and one focus group including 8 HCPs).

**Results:**

The workshop is student-centered and guided by an expert with an academic and clinical background. Learning was achieved with theoretical and practical components using authentic performance and collaborative learning. An active teaching and learning approach was used with strategies such as interactive lectures, hands-on elements, and role-playing (patient-counselor). The workshop emphasized an individual approach when prescribing PA integrating in clinical practice not only health benefits but also patient´s beliefs, motivations, needs, and barriers.

**Conclusions:**

Evidence-based practices and authentic performance were the most salient pedagogical elements used by EIM Colombia at the PA prescription workshop. A knowledge assessment that includes the practical aspect is suggested for future workshops. The curricular and pedagogical approach of the PA prescription workshop implemented in Colombia is well received by the medical community and a useful continuing medical education intervention with a potential contribution to current, and future health promotion needs.

**Supplementary Information:**

The online version contains supplementary material available at 10.1186/s12909-023-04999-3.

## Background

Global mortality from non-communicable diseases (NCDs) continues to reach unacceptable proportions [1]. Physical inactivity is an important risk factor adding to the NCDs pandemic [2] and is currently the fourth leading cause of death worldwide [3]. Moreover, sedentary behaviors have also emerged as potential risk factors for many chronic conditions and have increased morbidity and mortality [4]. In addition to improving the general health status and well-being of individuals, increasing physical activity (PA) levels has proven to be effective in both the prevention and treatment of NCDs at a minimal cost [5].

Healthcare professionals (HCPs) play a key role in promoting healthy habits, nonetheless, most practicing physicians lack the knowledge, skills, self-efficacy, and training to effectively do so [6, 7]. These counseling barriers are evident in the low rates of exercise counseling by physicians [8, 9]. Studies also suggest that physicians often miss the opportunities to counsel patients regarding the need for substantial lifestyle modifications at every clinic visit [10]. Therefore, it is necessary that HCPs develop competencies and use tools to effectively counsel patients regarding the benefits of the regular practice of PA [11].

Continuing medical education (CME) along with evidence-based practice is an effective approach to increase physicians’ knowledge and clinical competencies [12]. Most CME workshops include interactive and innovative techniques, which have been proven to be more compelling in fostering participants positive perceptions of the contents studied [13]. Therefore, a PA prescription workshop for physicians was developed by the Exercise is Medicine Regional Center in Latin America (EIM Latam) in 2010 and has been implemented since then [14]. This CME strategy has focused on educating HCPs in order to increase their knowledge and awareness about the benefits of PA, and provide practical tools to effectively prescribe exercise to their patients [15].

### EIM Latam PA prescription workshop

EIM Latam is part of the global initiative “Exercise is Medicine” (EIM) that was designed by the American College of Sports Medicine (ACSM) to promote PA at different societal levels, with a special emphasis in healthcare settings [16, 17]. EIM invites physicians to include PA as a vital sign in every patient visit and to deliver effective and personalized PA counselling and prescription. EIM has expanded globally and is now a multinational collaboration between North America, South America, Europe, Africa, Southeast Asia, China, and Australia [18]. The workshop has emphasized teaching primary care specialists and subspecialists to consolidate their knowledge and apply foundational concepts of PA (i.e. health benefits and risks associated with PA, screening and risk stratification and general principles of exercise prescription), as well as learn and practice how to perform a general fitness assessment and PA prescription as part of their clinical practice [14]. EIM Latam has delivered more than 140 workshops in 42 cities in 13 Latin American countries between 2011 and 2015. In total, 4854 HCPs have been trained on PA prescription in the region [15].

The collaboration between the EIM Research and Collaboration Center and the EIM Latam has showed that the one-day PA prescription workshop implemented in Latin America is effective in improving physicians’ knowledge and awareness on exercise prescription [19]. However, the pedagogical and curricular components implemented in the workshop have not been evaluated. Therefore, the aim of this study was to evaluate the curriculum and pedagogy proposed by EIM Latam in the PA prescription workshops implemented in Colombia between 2014 and 2015.

## Methods

### Study design and sample

This is a mixed methods study, with an explanatory sequential [20] design, in which the first phase is quantitative and the second one is qualitative. The purpose of this sequential approach is to draw integrated findings in the evaluation of the curriculum and pedagogy proposed by EIM Latam in twenty-six PA prescription workshops implemented in 9 cities of Colombia between 2014 and 2015. The workshop was free of charge to participants. Those scoring 80% or higher in the post-assessment received an international certification in exercise prescription endorsed by ACSM and EIM Latam [17]. These factors attracted physicians to attend the workshop, and applying an independent mixed methods sampling plan [21], a convenience sample, for the quantitative strand, and a retrospective sample of attendees, for the qualitative strand, were invited to participate. Quantitative methodology was used to determine participants’ health status, personal PA habits, and medical practice. At the end of the workshops, participants were also asked to fill out a survey about their satisfaction with the workshop. Qualitative methodology was used to better understand the curriculum of the workshop through four sources: 1. Analysis of the PA workshop manual of procedures, 2. Two workshops were observed, 3. Three semi-structured interviews were done to the program director and coordinators, 4. One focus group that included randomly selected participants was conducted.

The study received clearance and approval from the Ethical Review Committee of Universidad de los Andes (Act No. 539/2015). The participation in the study was voluntary, those who agreed to participate signed informed consent forms.

## Data collection and analysis

### Quantitative approach

Participants self-reported health status, personal PA habits and medical practice, as well as workshop satisfaction. A questionnaire was administrated at the beginning of the workshop to evaluate participant’s health status using a 4-point Likert scale, as follows: Excellent, good, average, and poor. Participants personal PA habits were evaluated using a validated short version of the International Physical Activity Questionnaire (i.e., meeting or not the global PA recommendation for health) [22]. Physicians reported frequency of PA assessment and prescription in their clinical practice using a 5-point Likert scale, as follows: Always, almost always, sometimes, or never. Lastly, role modeling and self-efficacy for PA prescription were reported using a 5-point Likert scale, as follows: Strongly agree, agree, neutral, disagree and strongly disagree. Out of the 1068 HCPs participants in the workshops, 795 returned the complete questionnaire (rate response 74,4%). The questionnaire lasted 15 min on average, questions can be found on supplementary material 1. Questionnaire for health care professionals. For analysis purposes, PA was calculated and classified in two categories based on the global PA recommendations for health (“met” or “not met”): meeting the PA recommendations was defined as participating in at least 150 min of PA of moderate intensity or 75 min of vigorous PA in the last seven days during leisure time [23]. We calculated the frequency of self-reported health status levels, meeting or not the global PA recommendations, assessing and prescribing PA and levels of agreements with role modeling and self-efficacy statements.

A survey administered at the end of the workshop was used to assess participant’s satisfaction with the workshop components, and the contribution to learning (i.e., pertinence of teaching contents, usefulness of given tools and impact on medical practice). Participants rated satisfaction using a 5-point Likert scale, as follows: very satisfied (4.6—5), satisfied (4.1—4.5), neutral (3.6—4), unsatisfied (3—3.5) and very unsatisfied (0—2.9). Among participants, 602 voluntarily filled out the satisfaction survey (rate response 56.4%). The survey lasted 3 min on average, questions can be found on supplementary material 2. Course evaluation.

Quantitative data collected were analyzed by descriptive statistics, including frequencies and percentages using Stata 16 V program.

### Qualitative approach

To evaluate the curricula and pedagogical approach, three of the five concurrent curriculum suggested by Posner were analyzed [24]: The official curriculum (described in formal documents), the operative curriculum (embodied in actual teaching practices and tests) and the hidden curriculum (institutional norms and values not openly acknowledged by teachers or institutions) [24]. Some guiding research questions for curricular analysis were: What are the purposes and content of the curriculum? How is it taught and how is it evaluated? what are the guiding principles and values of the curriculum? The data were collected from four sources:

1. *Manual of procedures analysis (official curriculum):* we analyzed the PA workshop manual of procedures, a document designed by EIM Latam in 2014 and implemented since then to effectively accomplish the academic and logistic standards of the workshop. The manual contains detailed instructions of the workshop methodology and can be consulted elsewhere [15].
2. *PA workshops observed (operative and hidden curriculum):* There were pre-scheduled PA workshops during the year 2015. Out of these, two workshops were chosen for independent observation by two trained curricular researchers, MTG and DG, who genuinely participated in the training, functioning solely as observers. Each researcher observed a separate workshop, with MTG observing in July and DG in October. The observation permitted the researchers not only to experience the workshop as a participant, but also to access participant’s dialogues and social interactions. Therefore, it allowed a detailed description of the observed phenomenon and understanding of the processes that took place in the workshop [25]. The observation began at 7:30 am and ended at 5:00 pm. The workshop observations were documented by a narrative into field notes. Field notes included information on data, resources, references, expressions, opinions, and facts that were of interest for the purpose of the study, it also considered feelings and thoughts generated by the observer as well as perceived critical incidents (concrete information on a specific subject of interest) [25].
3. *Semi-structured interviews (operative and hidden curriculum):* The semi-structured interviews were conducted with the EIM Latam director and two EIM Colombian workshop coordinators. A pre-formatted guide was used, including open-ended questions (Supplementary material 3. Interview 1). The interviews lasted 45 min on average. Study participants were able to express their own experiences and perspectives that allowed researchers to understand, describe and interpret the phenomenon studied [25].
4. *Focus group (operative, and hidden curriculum):* During a follow-up academic event, involving HCPs who had previously participated in a PA workshop in Colombia between 2014 and 2015, the focus group was conducted. 8 HCPs were randomly selected. Physicians signed a consent form and voluntarily agreed to participate in the focus group. The focus group followed a guided questionnaire and lasted 30 min on average (Supplementary material 4. Interview 2).

For analysis purposes, we used the constant comparative method [26]. Two members of the research group, a trained curricular researcher (DG) and a physical therapist trained in public health (DCP) separately identified common themes applicable to each concurrent curriculum. The analysis was conducted in Spanish. Some notes were translated into English by the bilingual research staff of Universidad de los Andes (DCP, MCA) to exemplify reports from workshop participants, the director, and coordinators. The method of analysis included the initial organization of the data, followed by the conceptualization and categorization using open, axial, and selective coding approaches. In case of discrepancies a third team member was required. We validated the identified common themes through a member validation process, also called member and expert checking [27], in which key informants (source of data during the data collection phase) and experts with extensive expertise on the area validated the identified information. As an important component of a mixed methods study, the integration of quantitative and qualitative strands was reached through the comprehensive multiperspective evaluation [28].

## Results

**Fig. 1:**
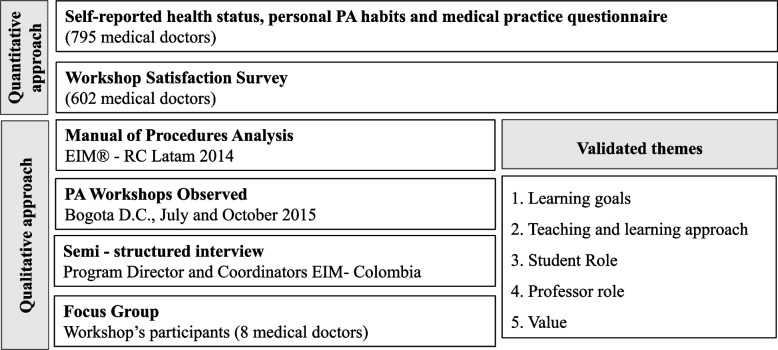
Mixed research methods to evaluate the curricula and pedagogical approach

For results interpretation, the mixed research methods used in this study provide a comprehensive understanding of the curriculum and pedagogy analysis, integrating the quantitative results with each qualitative validated them, including some relevant quotes from observation and key informants. The analysis of the qualitative data resulted in five themes after research validation [28]: learning goals, the teaching and learning approach, student role, professor role and values (Fig. 1).

### Quantitative analysis

#### Participant’s characteristics

Among the physicians participating in the PA workshops (*n* = 795) in Colombia, the majority were general practitioners (*n* = 304). The main medical specialties participating were pediatrics (*n* = 112), cardiology (*n* = 42), and family medicine (*n* = 31) (Table 1).

**Table 1:** PA Workshop participant´s characteristics

Medical specialties	Participants (%)
General practitioners	304 (38.2)
Pediatrics	112 (14.1)
Cardiology	42 (5.3)
Family medicine	31 (3.9)
Obstetrics	20 (2.5)
Internal medicine	19 (2.4)
Endocrinology	9 (1.1)
Sports medicine	8 (1.0)
Residents	5 (0.6)
Diabetology	4 (0.5)
Surgery	3 (0.4)
No data	2 (0.2)
Others^a^	241 (30.3)
**Total**	**795 (100)**

^a^Allergy, aerospace medicine, dermatology, epidemiology, gastroenterology, geriatrics, occupational medicine, physical medicine and rehabilitation, psychiatry, radiology, surgery. Subspecialties: Endocrinology, diabetology, cardiopulmonary rehabilitation. Health administration specialties: auditor, management, economy, public health

The vast majority (89%) reported to have a perception of good or excellent health. Half of them (51%) reported to currently engage in 150 min per week of moderate PA or 75 min per week of vigorous PA. Moreover, 80.2% and 87.2% reported to “always” or “almost always” evaluate or recommend PA to patients, respectively. Regarding medical practice, 93.9% “agreed” or “strongly agreed” with the statement “Physicians are responsible for promoting adequate PA levels” and 74.5% “agreed” or “strongly agreed” with the statement “I am effective in helping my patients to be physically active” (Table 2).

**Table 2:** Health status, PA habits and medical practice among HCPSs participating (*n* = 795)

**Participant´s answers (%)**	
Complies with global PA recommendations	
Yes	51.1
No	48.9
Health status	
Excellent	21.0
Good	68.2
Average	9.8
Poor	1.0
Evaluation of PA in their clinical practice	
Never	3.5
Sometimes	16.3
Almost always	33.2
Always	47.0
Recommendation of PA in their clinical practice	
Never	2.3
Sometimes	10.2
Almost always	34.2
Always	53.3
Agreement with:	
“Physicians are responsible for promoting adequate PA levels”	
Strongly disagree	3.6
Disagree	0.6
Neutral	1.8
Agree	21.7
Strongly agree	72.2
“I am effective in helping my patients to be physically active”	
Strongly disagree	2.9
Disagree	7.0
Neutral	15.7
Agree	46.0
Strongly agree	28.5

Participants evaluated the workshop contents and tools given. Overall, 93% of participants reported being “very satisfied” with the pertinence of teaching contents and 83% reported being “very satisfied” with the usefulness of the tools used (Table 3).

**Table 3:** Participants’ satisfaction with academic components and contribution to learning of the workshop

Category	Very dissatisfied	Dissatisfied	Neutral	Satisfied	Very satisfied
	**n (%)**	**n (%)**	**n (%)**	**n (%)**	**n (%)**
**Workshop Component**					
** (*n* = 602) Theoretical**	4 (0.7)	3 (0.5)	47 (7.8)	65 (10.8)	483 (80.2)
** (*n* = 602) Practical**	8 (1.3)	16 (2.7)	54 (9.0)	124 (20.6)	400 (66.5)
**Contribution to learning**					
** (*n* = 600) Pertinence **^**a**^	3 (0.5)	4 (0.7)	31 (5.2)	2 (0.3)	560 (93.3)
** (*n* = 592) Usefulness**^**b**^	5 (0.8)	12 (2.0)	79 (13.3)	3 (0.5)	496 (83.4)
**(*n* = 600) Impact**^**c**^	5 (0.8)	7 (1.2)	60 (10)	0 (0)	528 (88)
**Overall**	**3 (4)**	**4 (0.7)**	**23 (3.8)**	**101 (16.8)**	**471 (78.2)**

^**a**^Pertinence was obtained from the scoring average given by participants to the item *Pertinence of the topics*

^b^Usefulness was obtained from the scoring average given by participants to the item *Usefulness of the content according to your medical specialty*

^c^Impact was obtained from the scoring average given by participants to the item *Workshop impact on your medical practice*

## Qualitative analysis

### Curriculum and pedagogy approach

#### Learning objectives

The workshop objectives were clearly communicated to participants by the EIM Latam director and workshop coordinators at the beginning of the workshop, additionally they were documented within the manual of procedures.

*“1. Raise awareness about the importance of PA. 2. Provide medical evidence. 3. Provide tools in order to evaluate and prescribe PA appropriately” (Interview to EIM Latam director)*

The contents of the workshop were explained in lectures based on evidence-based medicine as analyzed in the manual of procedures and observed in the workshops. An important focus was given to cardiovascular risk factor screening, fitness assessment, promoting healthy behaviors and exercise prescription according to patient’s goals and expectations.

As was observed, the workshop also aimed to motivate HCPs to incorporate PA into their daily life. The director actively highlighted the importance of role modeling to effectively impart professional values, attitudes, and healthy behaviors to patients. The workshop gave insights to overcome challenges remaining on translating knowledge into practices to improve patient care. This was evident when talking to the participants during the focus group:

*“Many patients go running or biking with me (...). What the professor says is very true. You cannot give what you do not have! And if you don´t give your patients a good example, you can't be doing it right” (Focus group).*

#### Teaching and learning approach

The course has two components, one theoretical and one practical. The theoretical component included motivational and informative lectures based on an oral presentation. A workshop coordinator stated that most participants expressed to be surprised by the quantity and quality of evidence regarding PA. Motivational stories and personal experiences were shared by the director. One of the participants of the focal group stated:

*“I liked how PA was explained as a medicine. What if we see PA as drug that need to be prescribed? like a health product in the market” (Focus group)*

The practical component allows participants to consolidate their knowledge and apply all the theoretical principles. Physicians interacted with the tools given and played the roles of patient and physician, making it an active learning experience.

The educational approach created a horizontal classroom setting, contributing to the knowledge development by experiences and informal opinions, thus fostering collaborative learning. The workshop coordinator explained further how this was accomplished.

*“Participants are asked to prescribe PA to each other using the EIM prescription and referral format tool” (Workshop coordinator)*

#### Student’s role

The workshop was centered on the students, who took an active role when building new knowledge. Participants were involved into different learning experiences, to later be incorporated with patients. In this way, participants created their own perspectives and integrated the knowledge gained based on their needs. Students self-analyzed their health status and were aware of fitness markers such as blood pressure, weight and strength during the workshop. Expressions such as “I am too sedentary” or “I thought I was able to do this” were commonly mentioned by participants.

#### Director’s role

The main features that stand out of the director were his academic and professional authority, along with his inspirational and encouraging role model. These characteristics motivated participants from the beginning to the end of the workshop. This was exemplified in the following comment made by one workshop participant:

*“The professor has a unique ability to motivate us and find pertinent examples of how to overcome barriers when prescribing exercise” (Focus group)*

The director oriented participants with logical reasoning by using an organized learning environment in order to convey knowledge. Within the professor role, different aspects came into play such as humor, critical analysis, self-analysis, and comprehensive discussions regarding strategies to effectively counsel patients. Topics such as role modeling in medicine, industry influence and healthcare dynamics in Colombia were the most debated among participants.

*“I am sure that my patients have much more adherence because they see me as a fitness role model, in fact many patients go running or biking with me”*

#### Value

The workshop highlighted the importance of using an individual approach with patients when prescribing PA, considering their own motivations and needs. Participants recognized goal setting strategies such as the acronym SMART Goals (i.e., specific, measurable, achievable, realistic, time-bound) as an useful tool for PA counseling [29]. Likewise, the workshop added value to the PA prescription in clinical practice by integrating not only health benefits but also inherent characteristics or related personal beliefs such as physical appearance and physical functionality.

During the workshop, participants identified their own barriers to practice PA, and therefore, through a humanizing procedure, appreciated patient's barriers. One of the participants expressed her experience with patients:

*“Considering the strategies presented on the workshop, I would say that is very important to put yourself in you patient’s shoes to better understand their barriers” (Focus group)*

The medical specialty was a factor embedded within the hidden curriculum of the workshop. Some physicians consider PA prescription fundamental for patients’ health while others may be afraid of prescribing PA because of the absolute contraindications of exercise. The unresolved conflict was raised by participants in the focus group:

*“(...) when a patient goes to the cardiologist, he may be recommended to practice cardiovascular exercise, nonetheless, when he also goes to the orthopedist, he may be recommended to avoid exercise” (Focus Group)*

In addition, the workshop coordinators, and the director of EIM Latam reflected about the influence industry has on physician’s decision-making and value in health care delivery.

*“The industry has the perception that exercise prescription is a threat to economic growth and productivity, it may be because there are low-cost alternatives available to practice PA everywhere” (EIM Latam director).*

## Discussion

This study evaluated the curriculum and pedagogical strategy proposed by EIM Latam in the PA prescription workshops implemented in Colombia between 2014 and 2015. It was found that the curriculum and pedagogy strategy were consistent with the learning objectives of the workshop. The workshop methodology is student centered and the learning is achieved using authentic performance and collaborative learning. An active teaching–learning approach was used with strategies brought to context such as interactive lectures, hands on elements and role-playing. The importance of counselling patients individually according to unique characteristics and their knowledge was emphasized. These results underline the pertinence of the curriculum and pedagogical strategy proposed by EIM Latam to improve physician’s competencies to assess, counsel and prescribe PA to patients.

The learning goal of the workshop was to increase knowledge and awareness and provide useful and practical tools for HCPs to evaluate and prescribe PA in daily practice. Studies have shown limited physician training on PA [30]. This has led to a low proportion of medical PA counselling and prescription in clinical practice [9]. Similarly, CME have been reported to be an important starting point for addressing the lack of education regarding PA within the medical community [11, 31, 32].

The study found that 90% of participants reported having a good health status, but only half of them met recommendations on PA. This data is consistent with data found by Arciniegas Calle et al., in a Latin-American population, where it was reported that 53% of medical doctors comply with the aerobic PA recommendations and 38% with the resistance PA recommendations for optimal health [19]. Therefore, the workshop was also designed to motivate physicians to incorporate PA into their daily life through the practical component. It has been documented that when physicians adopt PA as a habit, they are more likely to encourage patients to adopt and maintain active behaviors [33]. Similar studies showed the relation between the accumulating recommended levels of PA of physicians and the higher levels of PA prescription performance [34, 35].

Half of the workshop´s participants indicated that they evaluate and recommend PA to their patients during medical consultation. However, the proportion in the general physician population could be lower, considering a selection bias in this study due to voluntary enrollment in the course. Similar educational experiences have reported that participating physicians already have a particular interest in PA prescription before attending a workshop [36]. In 2008, the National Survey of Energy Balance Related Care among Primary Care Physicians (PCPs) showed that less than a half of PCPs advise their patients to practice PA [37], in contrast with data from the National Health Interview Survey in 2010 that showed that 32% of patients report receiving exercise or PA guidance [8].

This workshop not only provided participants with knowledge, but also offered hands-on teaching elements. According to teaching adult principles, tools given to participants allowed them to improve retention and decision-making skills by applying immediately new knowledge and methods [38–40]. In addition, it has been demonstrated that CMEs that use practice sessions were positively correlated with change in professional practice [39, 40]. A similar study conducted in Canada found that attendees rated their likeliness to use the tools and resources provided in the workshop as high [41].

The role-playing in the workshop is a teaching and learning approach used by EIM Latam physicians to re-examine their fitness level. It is the opportunity to put themselves in someone else's shoes. An effective change in performance was observed in studies that used interactive techniques such as role-play or hands-on practice sessions [42]. The workshops promoted collaborative learning by allowing participants to share perspectives and learn from their own personal and professional experiences considering their medical background. Therefore, role-playing along with a collaborative learning approach contributed to reducing the existing gap between research and practice, as well as, enhancing participant’s empathy when prescribing PA [43].

Participant’s satisfaction with the theoretical and practical components of the course was very high, as reported in other EIM trainings within the global and Latin American contexts [41, 44]. However, some authors argue that a post course satisfaction survey is not a complete evaluation about learning and effectiveness of training in CMEs [45]. Despite this position, in our study the focus group and satisfaction surveys demonstrated coherent findings according to the workshop objectives and HCPs needs.

According to Biggs, assessment should reflect the learning goals as was found in this study [46]. Participant´s assessment focused on declarative knowledge, using a pre/post-test assessment. Indeed, some authors have considered this assessment model to have limitations evaluating physician's performance in clinical practice and the confounding factors influencing results [45]. However, in a previous study published by our research group, it was determined that all the participating specialties of the EIM Latam workshops had a significant increase in their knowledge especially in the topic of PA prescription, followed by PA benefits and risks, as well as the practical component which was included in the PA assessment [19]. The current assessment of the PA prescription workshop lacks the capacity to evaluate if participants are able to integrate their new knowledge into competences for their clinical practice. However, another study evaluating a 3-h workshop to increase PA prescription in family physicians assessed the effectiveness of intervention through a pre-post survey data, demonstrating an increase in proportion of reported PA prescriptions a month after intervention [34]. This might guide the effect of training in this topic; however, studies with a large follow-up and a specific framework of clinical performance are still needed [47].

Perrenoud emphasizes the need for professional teaching competences such as the ones seen in this study, including activities that organize and encourage learning, manage learning progression, engage students with continued medical education, and teamwork [48]. In addition, individualizing health care delivery and humanizing procedures through empathy is a significant learning process that is based on previous knowledge, beliefs, attitudes and connections to new learning [49]. It is key to understand the audience as well as the culture embedded in the medical profession for the workshop to be successful [50–52].

Furthermore, the workshop provided an essential opportunity to establish consistency in prescribing PA across diverse medical specialties with constructivist principles such as previous learning, collaborative learning, and student-centered learning [53]. Specialized training appears to better equip healthcare professionals to promote PA, with cardiologists playing a crucial role due to strong evidence supporting PA in cardiology for instance [42]. In this sense, disparities in PA prescription are heavily shaped by the presence or absence of public health policies that leads in varying levels of understanding and training regarding the role of PA in healthcare [42]. Indeed, addressing these multifaceted factors is essential to ensure patients consistently receive well-informed PA guidance, regardless of their medical specialty.

### Strengths and limitations

This is the first study aimed at analyzing the curricular and pedagogy approach for the PA prescription workshops used by EIM Latam. The research methods used in this study provided a more comprehensive understanding of the workshop, which could be used in future research, particularly related to CMEs strategies. Moreover, we ensured interviews were directly involved in the workshop and two researchers independently observed the PA workshop, which gave us diverse perspectives. Yet, a notable limitation is the self-reported nature of the data and recall bias that may have affected how participants recounted past experiences. While this study allowed us to study specifically the case of the EIM PA prescription workshop for physicians in Colombia, we cannot replicate these findings in the Latin American region, therefore, different contexts and actors need to be considered. Finally, it is important to evaluate whether physicians incorporate new knowledge into their clinical practices, but this was out of the scope of the study.

## Conclusion

Evidence-based practices and authentic performance were the most salient pedagogical elements used by EIM Colombia at the PA prescription workshop. A knowledge assessment that includes the practical aspect is suggested for future workshops. The curricular and pedagogical approach of the PA prescription workshop implemented in Colombia is well received by the medical community and is an effective continuing medical education intervention with potential contribution to current and future health promotion needs worldwide.

### Supplementary Information

**Additional file 1.**

**Additional file 2.**

**Additional file 3. **

**Additional file 4.**

## Data Availability

The data used and/or analyzed during the current study are available from the corresponding author on reasonable request.

## Abbreviations

PA: Physical activity
HCPs: Health care professionals
EIM: Exercise is medicine
EIM Latam: Exercise is medicine regional center in latin America
NCDs: Non-communicable diseases
CME: Continuing medical education
ACSM: American college of sports medicine American
PCPs: Primary care physicians
