# Supplementary material for: Curricular and pedagogical approaches for physical activity prescription training: a mixed-methods study of the “Exercise is Medicine” workshops in Colombia
Source: BMC Med Educ. 2024 Jan 22;24:79. doi: 10.1186/s12909-023-04999-3 (PMC10804704; doi:10.1186/s12909-023-04999-3)
Supplement: Supplementary file 1 — Additional file 1. [file 12909_2023_4999_MOESM1_ESM.docx]

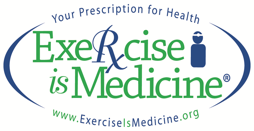


QUESTIONNAIRE FOR HEALTH CARE PROFESSIONALS

Dear Doctor, the questions below seek to collect information about your habits, lifestyle and medical practice. As part of the exercise prescription course and Exercise is Medicine® (EIM®) strategy, these data will be used to measure the impact of interventions performed by the EIM® Regional Center for Latin America on trained physicians. Therefore, and if you authorize, within six months you will receive a follow-up survey by email for your completion. The data collected will be obtained under a strictly confidential nature, and all personal information derived from it will be treated in accordance with Personal Data Protection Law 1581 of 2012 and Declaration of Helsinki. We remind you that your participation is completely VOLUNTARY and you can withdraw from the study at any time. If you have any concerns or additional information, you can write to us by email: eim.latam@gmail.com.

I declare that I have been informed about the study and use of the following data for research purposes. I agree to participate voluntarily:

Names ___________________________________

Email ____________________________________

Phone number __________________

Date ___________________ (DD/MM/YYYY)

City _______________ Country ________________

SOCIO-DEMOGRAPHIC VARIABLES

1. ¿ What is your date of birth? ___________________ (DD/MM/YYYY)
2. Your gender is (mark with an x):

a. Masculine b. Female

HEALTH CONDITIONS AND HABITS

1. What is your height in centimeters? __ __ __ cm
2. What is your weight in kilograms? __ __ __ kg;
3. Do you consider that in general your state of health is (mark with an x):
   - 1. Good b. Acceptable c. Poor d. Very Poor

We are interested in finding out about the kinds of physical activities that people do as part of their everyday lives. The questions will ask you about the time you spent being physically active in the last 7 days. Please answer each question even if you do not consider yourself to be an active person.

Please think about the activities you do at work, as part of your house and yard work, to get from place to place, and in your spare time for recreation, exercise or sport. Think about all the vigorous activities that you did in the last 7 days. Vigorous physical activities refer to activities that take hard physical effort and make you breathe much harder than normal. Think only about those physical activities that you did for at least 10 minutes at a time. 1.

1. During the last 7 days, on how many days did you do vigorous physical activities like heavy lifting, digging, aerobics, or fast bicycling?

_____ days per week

No vigorous physical activities (Skip to question 8).

1. How much time did you usually spend doing vigorous physical activities on one of those days?

_____ hours per day

_____ minutes per day

Don’t know/Not sure

Think about all the moderate activities that you did in the last 7 days. Moderate activities refer to activities that take moderate physical effort and make you breathe somewhat harder than normal. Think only about those physical activities that you did for at least 10 minutes at a time.

1. During the last 7 days, on how many days did you do moderate physical activities like carrying light loads, bicycling at a regular pace, or doubles tennis? Do not include walking. _____ days per week

No moderate physical activities (Skip to question 10)

1. How much time did you usually spend doing moderate physical activities on one of those days?

_____ hours per day

_____ minutes per day

Don’t know/Not sure

Think about activities intended solely for STRENGTHENING, such as lifting weights or doing other exercises to maintain muscle strength. Please include all these types of activities regardless of whether you have included them before.

1. During the last 7 days, on how many days did you perform muscle STRENGTHENING activities?

_____ days per week

No STRENGTHENING activities (Skip to question 12)

1. How much time did you usually spend doing muscle STRENGTHENING exercises on one of those days?

_____ hours per day

_____ minutes per day

Don’t know/Not sure

The last question is about the time you spent sitting on weekdays during the last 7 days. Include time spent at work, at home, while doing course work and during leisure time. This may include time spent sitting at a desk, visiting friends, reading, or sitting or lying down to watch television.

1. During the last 7 days, how much time did you spend sitting on a week day?

_____ hours per day

_____ minutes per day

Don’t know

HEALTH CARE PROFESSIONALS’ CHARACTERISTICS AND CLINICAL PRACTICE

1. Indicate your occupation (mark with an X all that apply):
2. General practitioner
3. Internal medicine doctor
4. Cardiologist
5. Endocrinologist
6. Pediatrician
7. Obstetrician/Gynecologist
8. Sport medicine physician
9. Other ____________
10. ¿ How often do you EVALUATE/ASK your patients about Physical Activity (PA)?
    1. Never
    2. Sometimes
    3. Very often
    4. Always
11. If in the previous question (#14) you answered “Sometimes”, “Very often” or “Always”, please specify how you EVALUATE/ASK your patients about PA (check all that apply):
12. General questions about the amount of AF
13. Specific questions about AF duration and intensity
14. Specific PA questions that are part of the electronic medical record
15. PA standardized questionnaire
16. Other (please specify) _______________________________
17. ¿ How often do you recommend PA to your patients?
    1. Never
    2. Sometimes
    3. Very often
    4. Always
18. If in the previous question (#16) you answered “Sometimes”, “Very often” or “Always”, please specify how you recommend PA to your patients (check all that apply):
19. Verbal counseling
20. Written prescription
21. Interconsultation with other health care professionals (specialist doctor, physiotherapist, trainer)
22. Referral to PA programs or fitness centers
23. Other (please specify) _______________________________

Indicate with your choice the degree of agreement or disagreement with the following statements:

|  | Strongly Disagree | Disagree | Undecided | Agree | Strongly Agree |
| --- | --- | --- | --- | --- | --- |
| "Doctors have the responsibility to promote adequate PA in their patients" |  |  |  |  |  |
| “If I exercise and am in good physical condition, I will have the ability to advise my patients in a more coherent and effective way” |  |  |  |  |  |
| “I am effective in helping my patients to  be physically active” |  |  |  |  |  |
