# Supplementary material for: Curricular and pedagogical approaches for physical activity prescription training: a mixed-methods study of the “Exercise is Medicine” workshops in Colombia
Source: BMC Med Educ. 2024 Jan 22;24:79. doi: 10.1186/s12909-023-04999-3 (PMC10804704; doi:10.1186/s12909-023-04999-3)
Supplement: Supplementary file 2 — Additional file 2. [file 12909_2023_4999_MOESM2_ESM.docx]

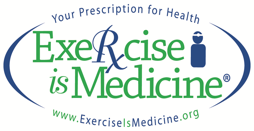


COURSE EVALUATION

Rate the following aspects of the delivered course from 1 to 5, with 5 being the best grade:

| Practicality of the content according to your medical specialty |  |
| --- | --- |
| Impact of the course on professional practice |  |
| Compliance with the schedule of activities |  |
| Facilities and general organization of the event |  |

Comments and suggestions:

________________________________________________________________________________

________________________________________________________________________________

________________________________________________________________________________

________________________________________________________________________________
