# Supplementary material for: Curricular and pedagogical approaches for physical activity prescription training: a mixed-methods study of the “Exercise is Medicine” workshops in Colombia
Source: BMC Med Educ. 2024 Jan 22;24:79. doi: 10.1186/s12909-023-04999-3 (PMC10804704; doi:10.1186/s12909-023-04999-3)
Supplement: Supplementary file 3 — Additional file 3. [file 12909_2023_4999_MOESM3_ESM.docx]

**INTERVIEW 1**

Objective: To investigate the understandings of the course designers.

Questions about curriculum design:

1. What do you seek to generate in those who take the course?

2. Under what criteria were the course contents chosen?

3. What medical education needs does the course seek to respond to?

4. What do you think is achieved with the activities proposed by the course?

5. What are the purposes of the course materials?

6. What are the reasons for structuring the learning assessment as the course has it?

Questions about the teacher's understandings:

7. In your opinion, what is the best way to learn?

8. What do you think should be the role of the teacher and what should be the role of the student in the course?

9. What do you feel that you have wanted to generate and have not been able to? Do you have any explanation for it?

10. What is your perception of the effectiveness of the course?

Questions about the hidden curriculum:

11. How high do you think is the level of acceptance of the central ideas of the course among those who take it? Because?

12. Do you perceive resistance to your proposals from your students or colleagues? What type?

13. How motivated do the participants arrive at the course? What do you think their motivations consist of?

14. What kind of values and skills other than the cognitive field do you think the course generates?

15. Do you feel that the course meets the expectations of the participants? Why?
