# Supplementary material for: Curricular and pedagogical approaches for physical activity prescription training: a mixed-methods study of the “Exercise is Medicine” workshops in Colombia
Source: BMC Med Educ. 2024 Jan 22;24:79. doi: 10.1186/s12909-023-04999-3 (PMC10804704; doi:10.1186/s12909-023-04999-3)
Supplement: Supplementary file 4 — Additional file 4. [file 12909_2023_4999_MOESM4_ESM.docx]

**INTERVIEW 2**

Objective: to investigate the understandings of those who have participated in the course.

1. How did you get to the course?

2. What motivated you to take this course?

3. What did you think you were going to do in the course before taking it?

4. What is your opinion about the contents of the course? Did they help you expand your understanding of the exercise prescription? Were they clear to you?

5. Do you feel that the course activities provided you with elements (knowledge, strategies, criteria) that you did not have before taking it? Like which ones?

6. In your opinion, what was the greatest contribution that the course gave you in your medical training?

7. What do you think the effect of the course has been on your professional practice? Have you stayed the same? Have you been transformed?

8. Have you used “screening” in patient care? Why? Have you encountered difficulties using it?

9. What kind of reasons do you give your patients to motivate them to exercise?

10. What could be the reasons why health care professionals do not insist on prescribing exercise? What is your opinion about it?

11. Do you feel that the course met your expectations in terms of the elements provided? Why?

12. Do you have any suggestions about any aspect of the course?
